# On being a *Hydra* with, and without, a nervous system: what do neurons add?

**DOI:** 10.1007/s10071-023-01816-8

**Published:** 2023-08-04

**Authors:** Alison Hanson

**Affiliations:** 1 Department of Biological Sciences, Neurotechnology Center, Columbia University, New York, NY USA; 2 Department of Psychiatry, New York State Psychiatric Institute, Columbia University, New York, NY USA

**Keywords:** Hydra, Neural evolution, Nerve-free

## Abstract

The small freshwater cnidarian *Hydra* has been the subject of scientific inquiry for over 300 years due to its remarkable regenerative capacities and apparent immortality. More recently, *Hydra* has been recognized as an excellent model system within neuroscience because of its small size, transparency, and simple nervous system, which allow high-resolution imaging of its entire nerve net while behaving. In less than a decade, studies of *Hydra’s* nervous system have yielded insights into the activity of neural circuits in vivo unobtainable in most other animals. In addition to these unique attributes, there is yet another lesser-known feature of *Hydra* that makes it even more intriguing: it does not require its neural hardware to live. The extraordinary ability to survive the removal and replacement of its entire nervous system makes *Hydra* uniquely suited to address the question of what neurons add to an extant organism. Here, I will review what early work on nerve-free *Hydra* reveals about the potential role of the nervous system in these animals and point towards future directions for this work.

## Introduction

How and why the first nervous systems evolved remain deep, unanswered questions. Because nervous systems likely emerged around 560 million years ago (Budd and Jensen 2017), we must rely on indirect information from fossils and extant organisms to infer when and how neuron-based integrative systems may have evolved and what new capacities they might have afforded non-neural animals. Traditionally, these questions mostly have been addressed with phylogenetic studies, where the neurological “parts list” (genes, molecules) from different extant phyla are compared to determine when and where certain neural components appeared, and hypothesizing about what new function each part might have bestowed upon the organism (Burkhardt and Jékely 2021). While this general approach has taught us much, to directly address the question of what a nervous system contributes to an organism, ideally one could both add and remove that system from a living animal and observe the resultant effects. Fortunately, evolution has left us with at least one such organism in which this remarkable feat is possible—the small freshwater polyp, *Hydra*.

**Fig. 1:**
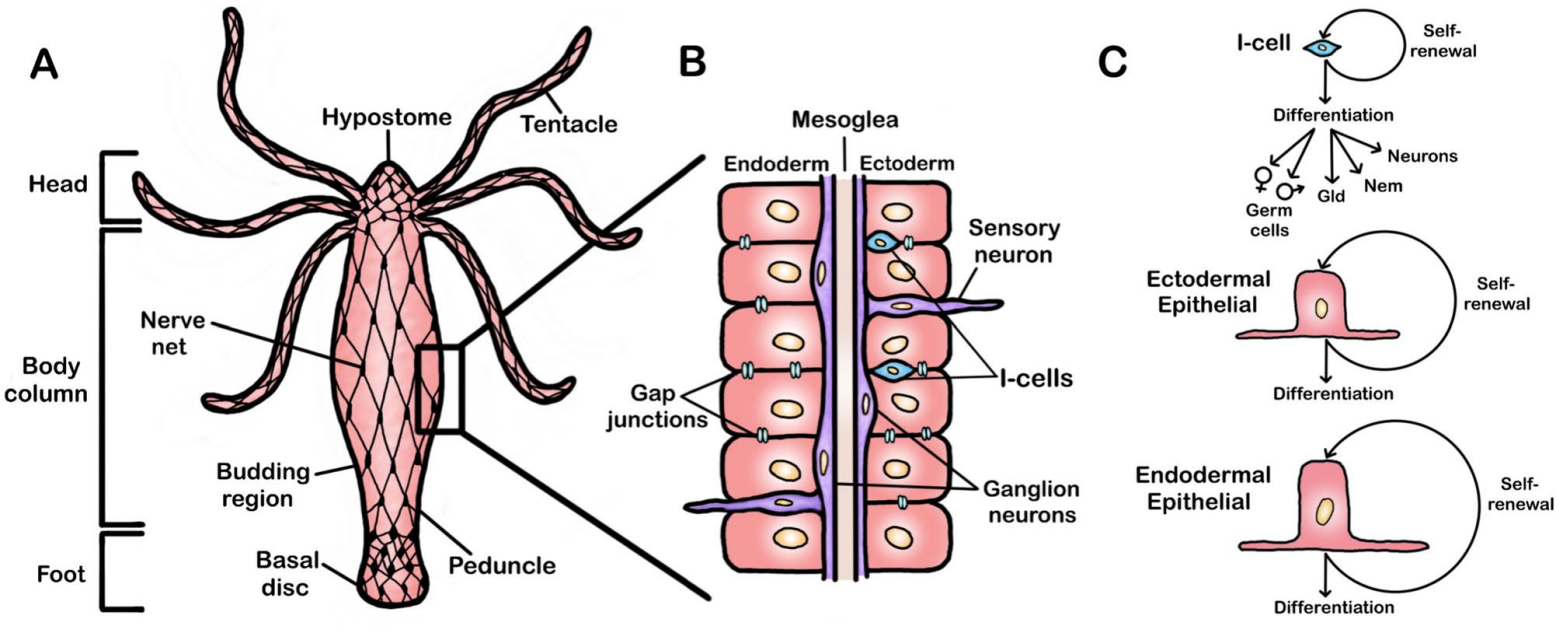
*Hydra* anatomy. **A** The basic body plan of *Hydra.* **B** The two tissue layers of *Hydra* are depicted with endodermal epithelial cells on the interior, ectodermal epithelial cells on the exterior, and an acellular mesoglea in between. Ganglion neurons are found at the base of the epitheliomuscular cells while sensory neurons protrude from both the endoderm and ectoderm. Interstitial-cells (I-cells) are located between ectodermal epithelial cells. Not shown are gap junctions between extensions of the endodermal and ectodermal epithelial cells within the mesoglea. **C** The three stem cell lineages of *Hydra* are shown with the unipotent endodermal and ectodermal epithelial cells and multi-potent I-cells that produce four cell types: germ cells, gland cells (Gld), nematocytes (Nem), and neurons

*Hydra* is a metazoan belonging to the phylum Cnidaria (Glauber et al. 2010), the sister group to all bilaterians, and possesses one of the most “primitive” known nervous systems in the form of a diffusely distributed nerve net (Hadzi 1909; Burnett and Diehl 1964; Lentz and Barrnett 1965; Lentz 1968; Dupre and Yuste 2017) (Fig. 1A). The *Hydra* body is a tubular structure composed of two tissue layers: the ectodermal epithelium on the outside and endodermal epithelium on the inside, with an acellular layer, the mesoglea, sandwiched in between (Campbell and Bode 1983; Glauber et al. 2010) (Fig. 1B). Three cell lineages continually create the *Hydra* body: ectodermal epithelial cells, endodermal epithelial cells, and interstitial stem cells (i-cells) (Fig. 1C). The ectodermal and endodermal epithelial cells are both fully differentiated epitheliomuscular cells that also act as stem cells in the body column where they continually self-renew (David and Campbell 1972; Buzgariu et al. 2015). As the epitheliomuscular cells are displaced to the apical or basal poles of the animal, they stop cycling and terminally differentiate (Campbell 1967; Dübel et al. 1987).

The i-cells are located in between the ectodermal epithelial cells and function as multipotent stem cells that give rise to four cell types: neurons, nematocytes (stinging cells), gland cells, and germ cells (David and Gierer 1974; David and Murphy 1977; Campbell and Bode 1983; Bode 1996). Importantly, each of the three cell lineages appear to be truly independent, with no evidence of any lineage being able to replace another (Marcum and Campbell 1978a; Buzgariu et al. 2015). For example, if the i-cells of an animal are removed, neither the ectodermal or endodermal epithelial cells can replace the i-cells or their products (Marcum and Campbell 1978a). Given that i-cells produce all neurons, if the i-cells of the animal are removed, so too are the neurons and nerve net they continually construct. Thus, if a method existed by which i-cells of *Hydra* could be removed without disrupting the ectodermal or endodermal epithelial cells, a “nerve-free” *Hydra* consisting of only epithelial cells could be created.

Early attempts at specifically eliminating i-cells from *Hydra* employed X-irradiation (Brien and Reniers-Decoen 1955), γ-irradiation (Clarkson and Wolpert 1967), and nitrogen mustard (Burnett and Diehl 1964), all of which significantly damaged epithelial cells in addition to i-cells, making it difficult to interpret results. It wasn’t until the late 1970’s that several methods for specifically eliminating i-cells were discovered, including chemical treatments (colchicine (Campbell 1976), hydroxyurea (Bode et al. 1976; Sacks and Davis 1979)), genetic manipulation (Sugiyama and Fujisawa 1978a; Terada et al. 1988), and low-dose γ-irradiation (Fradkin et al. 1978). Colchicine induces phagocytosis of i-cells by endodermal epithelial cells via an unknown mechanism (Campbell 1976), while hydroxyurea kills i-cells in the S-phase of the cell cycle (Bode et al. 1976). The thermosensitive *Hydra* strain (Sf-1) eliminates i-cells after heat-shock (Terada et al. 1988), and low-dose γ-irradiation induces DNA damage in i-cells (Fradkin et al. 1978).

**Fig. 2:**
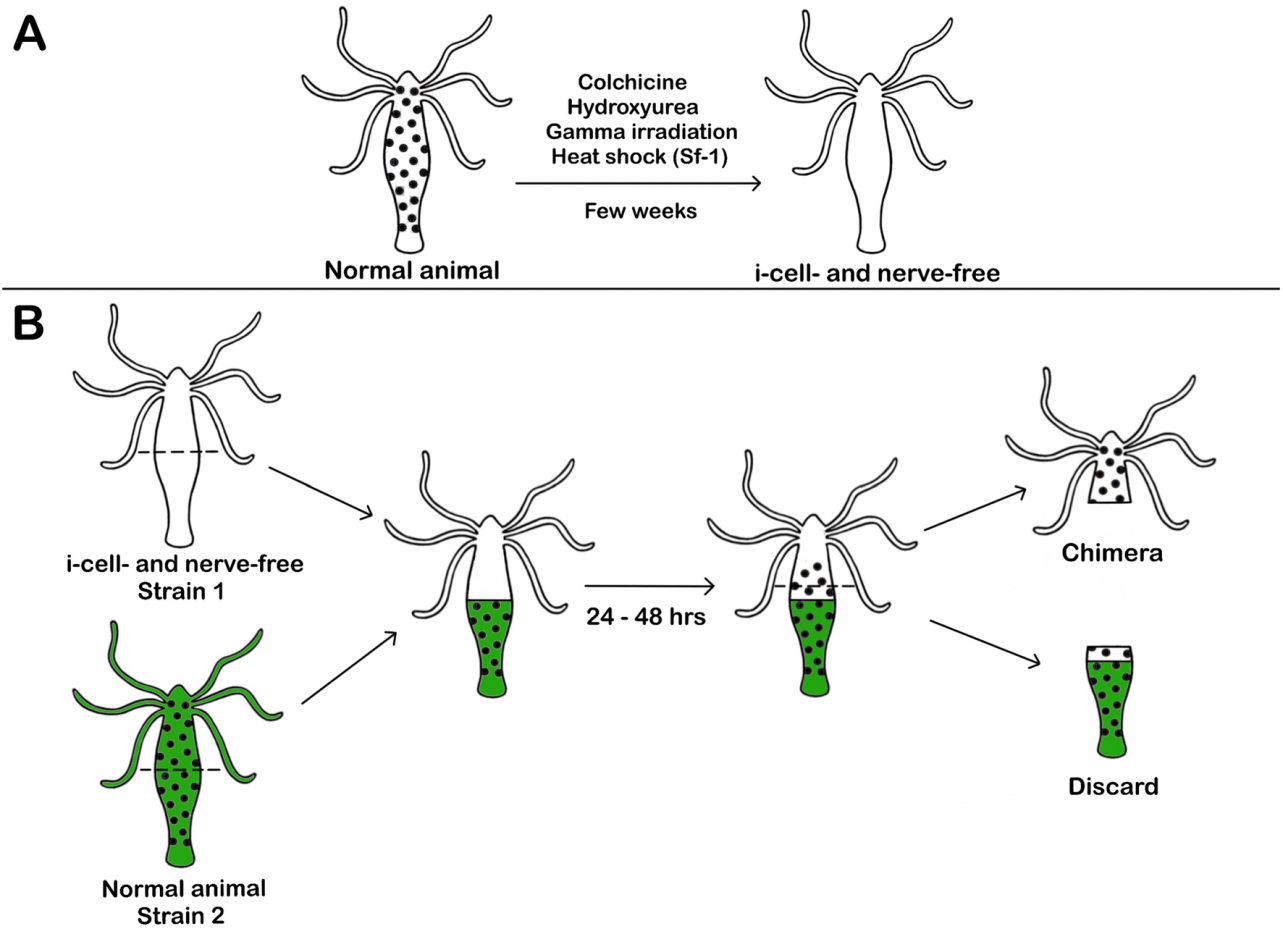
Removal and replacement of interstitial cells of *Hydra*. **A** Treatment of normal *Hydra* with colchicine (Campbell 1976), hydroxyurea (Bode et al. 1976), and gamma irradiation (Fradkin et al. 1978) results in an interstitial cell- (i-cell) and nerve-free animal after several weeks. The genetically modified strain, Sf-1, also produces an i-cell- and nerve-free animal several weeks after heat shock (Sugiyama and Fujisawa 1978a; Terada et al. 1988). **B** To replace i-cells, the top half of an i-cell- and nerve-free animal is grafted onto the bottom half of a vitally stained normal animal of the same, or different, strain. The two halves remain grafted for 24–48 h to allow the i-cells to migrate from the normal tissue into the i-cell- and nerve-free tissue. After 24–48 h, the bottom half of the animal is cut off and the top half of the animal consisting of the original i-cell- and nerve-free tissue with donor i-cells is allowed to regenerate, forming a new nerve net derived from the donor i-cells (Saffitz et al. 1972; Marcum and Campbell 1978a, b; Sugiyama and Fujisawa 1978b; Lee and Campbell 1979). Black dots represent i-cells

Regardless of the method used to eliminate i-cells, it takes several weeks for the animal to become “nerve-free” as its terminally differentiated neurons are gradually lost through tissue displacement (Fig. 2A). After several weeks, however, each method produces a surprisingly viable animal consisting of only ectodermal and endodermal epithelial cells and no i-cells or neurons (Bode et al. 1976; Campbell 1976; Fradkin et al. 1978; Sacks and Davis 1979; Terada et al. 1988). It is important to note these animals are also devoid of nematocytes (stinging cells), gland cells, and germ cells as i-cells also produce those cell types. Thus, these methods eliminate all cell types derived from i-cells, not just neurons. Once neurons have been removed from *Hydra*, it is then possible to perform a “nervous system transplantation” and add them back (Saffitz et al. 1972; Marcum and Campbell 1978b; Sugiyama and Fujisawa 1978b; Lee and Campbell 1979). This is done by grafting normal *Hydra* tissue of either the same, or a different, strain onto the nerve-free animal and allowing the i-cells to migrate into the nerve-free tissue (Fig. 2B). Once the i-cells have been “transplanted” into the nerve-free tissue, they can re-create a new nerve net composed of neurons from either the same, or a different, *Hydra* strain.

The astonishing ability to both remove and replace an entire nervous system in a living animal makes *Hydra* uniquely suited to address the question of what neurons *currently* add to the organism. Traditionally, nervous systems are often thought to have arisen to coordinate behavior and *move* multicellular bodies (Parker 1919; Mackie 1970, 1990; Keijzer et al. 2013; Jékely et al. 2015). However, these early nerve-free studies in *Hydra* indicate its nerve net is likely playing many other non-canonical roles. While most studies were performed decades ago with outmoded methods, they still have much to teach us about what kind of animal exists both with and without a nervous system. That is, what kind of body, sensation, information integration, behavior, learning, and memory does each type of animal possess? In short: what does a nervous system add to a living animal, here, now? I will first review what the earlier work with nerve-free *Hydra* has revealed about the potential role of the nervous system in the various processes outlined above. I will then summarize these findings and discuss what they imply about the overall role of the “primitive” nerve net in *Hydra*. The review will end with a discussion of future directions for this work using modern methods.

## What kind of animal exists with and without a nervous system?

As mentioned, nerve-free *Hydra* research reached its pinnacle in the late 1970’s after the first breakthrough by Richard Campbell, who discovered the double colchicine method for eliminating i-cells (Campbell 1976). This discovery was quickly followed by three other methods (hydroxyurea (Bode et al. 1976; Sacks and Davis 1979), genetic manipulation (Sugiyama and Fujisawa 1978a; Terada et al. 1988), and low-dose γ-irradiation (Fradkin et al. 1978)) also capable of producing viable nerve-free *Hydra*. Importantly, no matter which method was used to remove i-cells, each study produced essentially the same results, indicating the findings were due to loss of i-cells, not an artifact of any particular method. Here, I will summarize the findings of these studies and what they tell us about what kind of *Hydra* exists both with and without i-cells. For simplicity I will use the term “nerve-free” when talking about i-cell-free animals, keeping in mind these animals also lack nematocytes, gland cells, and germ cells.

### What kind of body?

To begin, what kind of *Hydra* body gets built and how is it maintained with and without neurons? This will include a discussion of differences in morphology, development, and regeneration in normal and nerve-free animals and what these differences can tell us about the potential role of the nervous system in these processes.

**Fig. 3:**
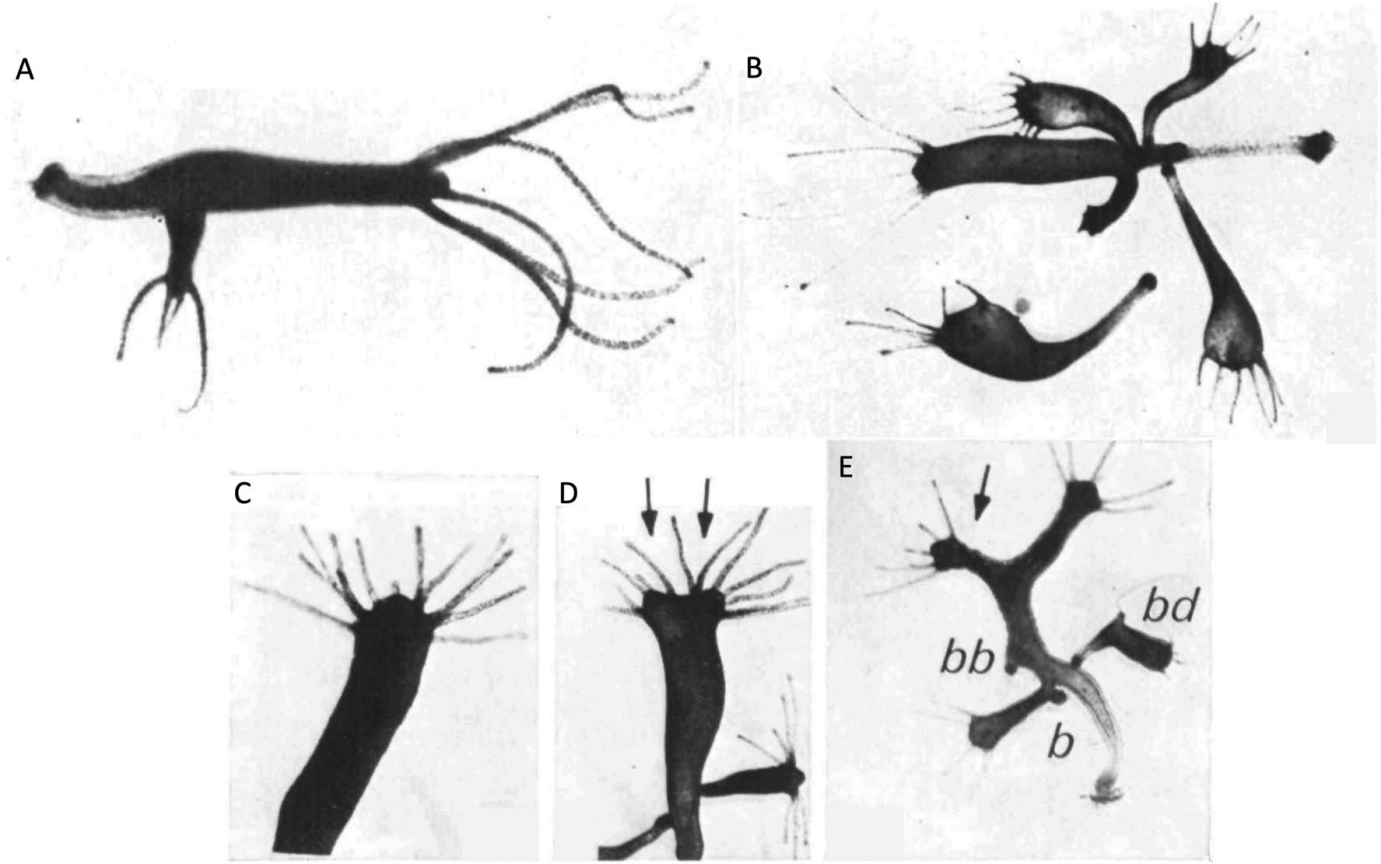
Morphology of *Hydra* with and without neurons. **A** Normal *Hydra* with characteristic morphology, including a dome shaped hypostome, long regularly spaced tentacles of equal length, narrow body column, and one attached bud. **B** Nerve-free *Hydra* produced by the double colchicine method with abnormal morphology, including irregularly spaced, short, straight, skinny, supernumerary tentacles of variable lengths; large, flat hypostomes; swollen gastric regions; narrow long peduncles; small basal discs; and multiple attached buds. A detached bud with the same abnormal morphology is on the bottom left. Nerve-free *Hydra* produced by the hydroxyurea method also shows abnormal numbers of irregularly spaced, short, straight, skinny, supernumerary tentacles of variable lengths **C-E**; animals with two heads (arrows) **D-E**; and animals with secondary basal discs (bb), buds attached via their peduncles instead of their basal discs (b), and buds with secondary tentacles off the sides of their bodies (bd) **E**. A and B adapted with permission from Fig. 1A and G, respectively in (Campbell 1976). C–E adapted with permission from Fig. 2A–C, respectively in (Sacks and Davis 1979)

As shown in Figs. 1A and 3A, normal adult *Hydra* possess a tubular body with a dome-shaped hypostome (head) with long equal length tentacles, narrow body column, peduncle (stalk), basal disc (foot), and budding region above the peduncle where typically 2–4 buds are attached in a budding animal (Campbell and Bode 1983). Regardless of the method used, when i-cells (thus neurons) are removed, the morphology of the body is significantly altered (Fig. 3B), resulting in the following abnormalities: irregularly spaced, short, straight, skinny, supernumerary tentacles of variable lengths devoid of nematocytes; large, flat hypostomes; swollen gastric regions; narrow longer peduncles; small basal discs; and buds that remain attached to parents for days or weeks resulting in animals with up to 6 or 7 attached buds (Campbell 1976; Fradkin et al. 1978; Marcum and Campbell 1978a; Sugiyama and Fujisawa 1978a; Sacks and Davis 1979). The hypostomes in i-cell-free animals may be flat, in part, due to loss of gland cells (i-cell products) that are known to be concentrated in the sub-hypostomal region of normal *Hydra* (Wood 1979). A significant number of nerve-free animals also possess more striking abnormalities such as secondary hypostomes and basal discs, and buds that are attached to their parents via their peduncles instead of their basal discs (Sacks and Davis 1979) (Fig. 3C–E). Importantly, when i-cells (thus neurons and nematocytes) are added back to the animal via grafting (Fig. 2B), the morphology of the animal is rescued and returns to normal (Fradkin et al. 1978; Marcum and Campbell 1978a; Sugiyama and Fujisawa 1978a). This suggests the epithelial cells were not damaged by the methods used and that i-cells (and their products, including neurons) play a critical role in creating and maintaining the normal *Hydra* body.

Nerve-free animals also show abnormalities in various developmental processes, including growth and budding rates, induction, regeneration, and maintenance and reversal of tissue polarity. Nerve-free *Hydra* initially grow and bud at a faster rate than normal animals if they are hand fed (this will be discussed further in Sect. “What kind of behavior?” below), but after several months, growth and budding rates significantly decline and start to exhibit “erratic” patterns where the growth rate stabilizes for a period of time then sporadically increases again (Sacks and Davis 1979). Nerve-free buds also remain attached to the parent much longer than normal animals do and often do not detach (Fig. 3B), leading to the formation of secondary hypostomes (Campbell 1976; Marcum and Campbell 1978a; Sugiyama and Fujisawa 1978a; Sacks and Davis 1979) (Fig. 3D–E). When i-cells (thus neurons) are added back to these animals, they resume normal growth and budding rates (Marcum and Campbell 1978a; Sugiyama and Fujisawa 1978a), suggesting i-cells (and their products, including neurons) play a role in regulating these processes.

Normal apical and basal *Hydra* tissue has strong inductive properties (Browne 1909; Webster 1971). That is, if hypostomal (head) tissue of *Hydra* is transplanted onto the body column of another *Hydra*, a second axis (head) will be induced, whereas if foot tissue is transplanted onto the body column, a second foot will be induced. If body column tissue is grafted onto the body column of another *Hydra*, no induction will occur and it will be resorbed. These same capacities were tested in nerve-free *Hydra* with unusual findings (Marcum and Campbell 1978a). Most often, the expected results were obtained. There were, however, some noticeable outliers. When transplanting normal hypostomal tissue onto the body column of a nerve-free animal, two out of five animals did not produce a second axis. In the same study, when normal body column tissue was grafted onto a nerve-free body column, a second axis was induced in one out of five animals. In a separate study (Sugiyama and Fujisawa 1978a), when either normal or nerve-free hypostomal tissue was grafted onto the hypostome of a nerve-free animal, instead of the tissue being resorbed (as normal), a foot was formed. Additionally, when either normal or nerve-free hypostomal tissue was grafted onto the foot of a nerve-free animal, the tissue was resorbed instead of forming a second axis (as normal). Thus, while nerve-free *Hydra* seem to maintain most inductive capacities, they also exhibit some interesting, unexplained abnormalities.

Nerve-free *Hydra* also display abnormal regenerative capacities. While nerve-free animals can regenerate a head and foot and maintain proper polarity like normal *Hydra*, they do so more slowly and less precisely (Marcum and Campbell 1978a; Sacks and Davis 1979; Miljkovic-Licina et al. 2007). The heads regenerated in nerve-free animals take a day longer to form and exhibit more variance in tentacle number, length, and spacing, as seen in the morphology of the adult animals described above (Sacks and Davis 1979; Miljkovic-Licina et al. 2007) (Fig. 3B–E). One of the most stringent tests of regenerative capacity is the ability of the animal to reverse its polarity in “polarity reversal” experiments (Marcum et al. 1977). Here, the head and foot are cut off the body column of one animal leaving just the body column. Then, a vitally stained foot from another animal is grafted onto the head end of the body column while a vitally stained head is grafted onto the foot end of the same body column such that the head and foot ends of the original body column have been reversed. At 12-h intervals the grafted tissue is removed and the original body column is allowed to regenerate. The resulting animals are scored in terms of their final polarity. Three possibilities exist: no polarity reversal (the head and foot regenerate in the same orientation as the original body column), mixed polarity (heads form at both ends or in the middle of the body column), or reversed polarity (the head and foot regenerate in reverse orientation from the original body column). When put to this test, nerve-free *Hydra* were surprisingly able to reverse their polarity with essentially the same kinetics as normal animals, but only rarely produced mixed polarity animals that are typically found with normal *Hydra* (Marcum et al. 1977). Thus, again, the nerve-free animals performed nearly, but not precisely, like normal *Hydra*.

Overall, these initial studies were quite shocking as they revealed *Hydra* can build and maintain its body quite well without neurons. While not normal in all aspects, the differences in morphogenesis of the nerve-free animals were mostly quantitative, not qualitative. This challenged the prevailing view at the time in which neurons were thought to be essential for *Hydra* development by secreting critical morphogens (Schaller et al. 1996). Given these surprising results, a series of chimera experiments were performed to further clarify whether the epithelial cells or the i-cells (and their products, including neurons) ultimately control *Hydra* development (Marcum and Campbell 1978b; Sugiyama and Fujisawa 1978b; Lee and Campbell 1979). This was done by transplanting the i-cells of one *Hydra* strain into nerve-free *Hydra* of another strain (see Fig. 2B) and determining whether the resulting chimeric animal resembled the i-cell or epithelial cell strain. The results were equivocal, leading to the conclusion that epithelial cells and i-cells must jointly control *Hydra* development with epithelial cells likely functioning as the mechanical components, or “effectors,” which are normally “patterned” by neurons (Marcum and Campbell 1978b). How neurons might pattern epithelial cells during *Hydra* development will be discussed in more detail in the discussion section below.

### What kind of sensation?

**Fig. 4:**
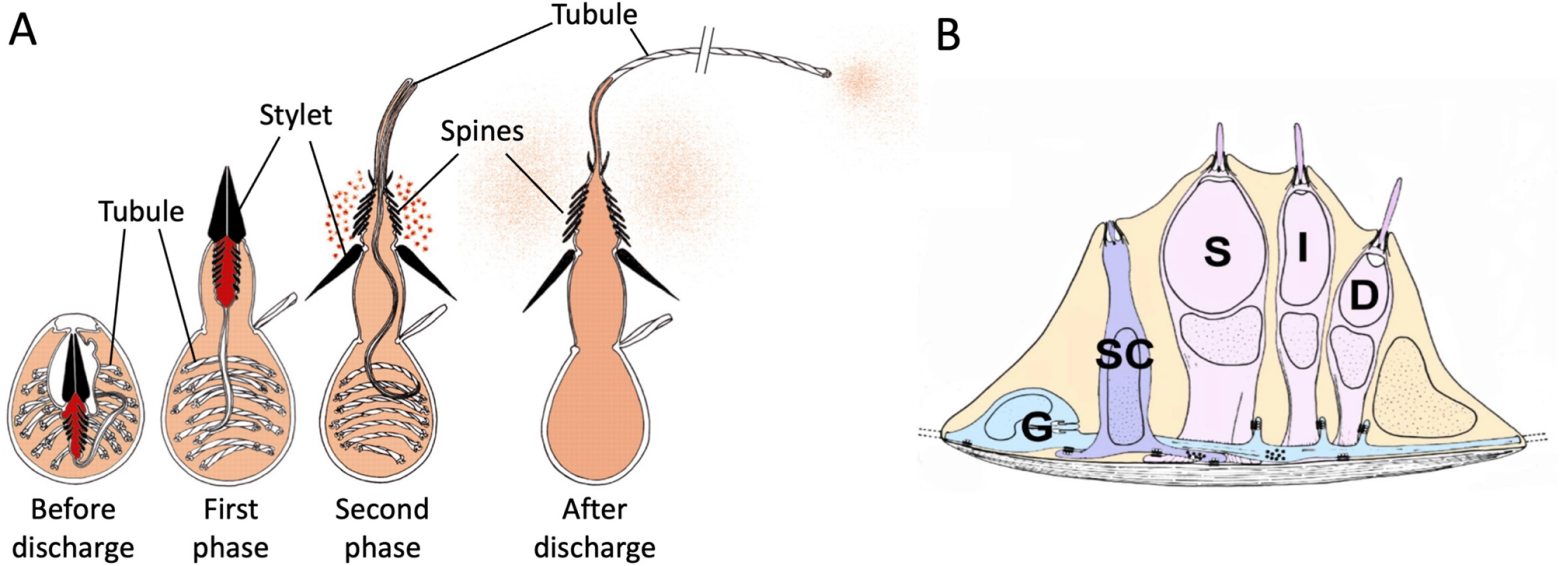
Nematocyte and battery cell complex anatomy. **A** Schematic of the stenotele nematocyte discharge process. Mechanical and chemical stimulation of the cnidocil on the nematocyte cell surface (not pictured) causes nematocyst discharge in two phases. In the first phase, the stylet is rapidly ejected. In the second phase, the stylet opens and spines and tubule are fully ejected. Figure adapted with permission from (Szczepanek et al. 2002). **B** Diagram of battery cell complex with cnidocytes and neurons embedded in a giant epitheliomuscular cell. Stenotele cnidocytes (S) are found in the center surrounded by isorhiza (I) and desmoneme (D) cnidocytes and neural sensory cells (SC), which form synapses with neural ganglion cells (G) that also connect all cnidocytes. Figure adapted from Fig. 1A in (Plachetzki et al. 2012) (http://creativecommons.org/licenses/by/4.0/)

Normal *Hydra* perceive light (Passano and McCullough 1962, 1965; Plachetzki et al. 2012), chemicals (Ewer and Fox 1947; Loomis 1955; Lenhoff 1961), mechanical stimulation (Mast 1903; Wagner 1905; Rushforth et al. 1963; Badhiwala et al. 2021), temperature (Mast 1903; Schroeder and Callaghan 1981; Bosch et al. 1988; Tzouanas et al. 2021), and gravity (Ewer 1946). While the precise mechanisms of most of these sensory modalities are not known, two main cell types are thought to be involved: sensory neurons and nematocytes, both derived from i-cells. Nematocytes or cnidocytes (stinging cells) are unique to Cnidaria and are one of the most complex cell types known (Hessinger and Lenhoff 1988; Kass-Simon and Scappaticci 2002). There are four types of nematocytes in *Hydra* (stenotele, desmoneme, and atrichous and holotrichous isorhiza) (Hessinger and Lenhoff 1988). Each nematocyte contains a different kind of encapsulated cyst (nematocyst) that is filled with barbed tubules and other substances (e.g., toxins), depending on the type of nematocyst. Upon either mechanical or chemical stimulation of hair-like projections (cnidocils) on the nematocyte membrane, nematocysts are discharged for defense, prey capture, or locomotion (Fig. 4A) (Hufnagel et al. 1985; Kass-Simon and Scappaticci 2002). Nematocytes are most highly concentrated in “battery cell complexes” in the tentacles of the animal where the four different nematocyte types, along with sensory and ganglion neurons, are embedded in a large epitheliomuscular cell (Hufnagel et al. 1985; Hufnagel and Kass-Simon 1988; Kass-Simon and Scappaticci 2002) (Fig. 4B). It is thought nematocytes directly sense their external environment via mechanoreceptors and chemoreceptors on their cnidocil, in addition to receiving input from surrounding sensory neurons (Kass-Simon 1988; Scappaticci et al. 2010). The battery cell complexes are thought to be the primary environmental sensors for the organism, but nematocytes and sensory neurons are also found throughout the *Hydra* body. Nematocytes are located almost exclusively in the ectoderm (Anderson and Bouchard 2009), while sensory neurons protrude from both the ectoderm and endoderm of the animal to sense the external and gastric environments, respectively (Lentz and Barrnett 1965).

The most well-studied sensory modalities in *Hydra* are mechanosensation and chemosensation leading to prey capture. Both sensory neurons and nematocytes are thought to be involved in this process whereby mechanoreceptors on both cell types respond to mechanical stimulation by moving prey (Anderson and Bouchard 2009; Scappaticci et al. 2010). The mechanoreceptor response can be sensitized by the presence of chemicals released by wounded prey, such as reduced glutathione, which can alter the threshold for triggering nematocyst discharge (Watson and Hessinger 1989; Scappaticci et al. 2010). More recently, light has also been shown to regulate the firing of nematocytes (Plachetzki et al. 2012). Phototaxis towards light had been well-established in *Hydra* since its original discovery by Trembley (Trembley et al. 1744; Wilson 1891), but how an animal without eyes was able to detect light remained a mystery until 2012, when an opsin (*Hmops2*) was shown to be expressed in sensory neurons of battery cell complexes (Plachetzki et al. 2012). In addition to opsin, co-expression of other phototransduction components found in bilaterians (e.g., cyclic nucleotide gated ion channel (Plachetzki et al. 2010) and Arrestin (Dolph et al. 1993; Krupnick et al. 1994)) was demonstrated, suggesting the basic machinery for converting light into an electric signal is highly conserved. Ultimately, this work demonstrated nematocytes fired more under dim versus bright light, which was hypothesized to be due to prey casting shadows over the tentacles of the animal (Plachetzki et al. 2012).

Altogether, it seems the battery cell complexes lining the tentacles may be able to receive and integrate mechanical, chemical, and light information to tightly regulate the discharge of nematocytes, which are energetically expensive single-use cells (Anderson and Bouchard 2009). In addition to mechanosensation in its tentacles leading to prey capture, *Hydra* also displays a robust response to mechanical stimulation along its entire body column, although it does exhibit some regional specificity (Badhiwala et al. 2021). If the foot is removed, no significant difference in mechanosensation is observed, whereas removal of the head results in a significant reduction in mechanosensory response. If both head and foot are removed, mechanosensation is even more significantly reduced, but not absent. These results suggest *Hydra* is mechanosensitive throughout its body but requires both its head and foot for maximum mechanosensory response. Which mechanoreceptors and what cell types participate in this process are unknown.

*Hydra* is also thermosensitive (Tzouanas et al. 2021). It responds to acute positive changes in temperature by first elongating then contracting in a reproducible manner. It is not known why *Hydra* exhibits this particular thermosensitive response. The thermoreceptors and cell types contributing to this response also remain to be discovered. Finally, there is evidence from one early study that *Hydra* also senses gravity (Ewer 1946). While observing a tank of *Hydra* in his classroom, Ewer noticed all buds would migrate up the side of the tank to the surface of the water. He determined this was a gravity response, not due to effects of oxygen, carbon dioxide, or pH. To date, no evidence of a gravity sensing organ, or statocyst, has been reported in *Hydra,* although an unusual statocyst was discovered in the Hydrozoan *Corymorpha palma* (Campbell 1972).

As can be seen, normal *Hydra*, with its full complement of neurons and nematocytes, has a very rich sensory repertoire providing it with access to an extensive outside world—one in which it can effectively “see,” “taste,” “smell,” “touch,” detect temperature, and even sense “up” from “down.” What kind of sensory world remains for *Hydra* without i-cells (thus neurons)? Only one early study has addressed this question (Campbell et al. 1976). Nerve-free *Hydra* was found to no longer respond to bright light, shrimp extract, or mild mechanical stimulation. It *did* respond to both strong mechanical stimulation and direct electrical stimulation of the body column (Campbell et al. 1976), however, suggesting some mechanosensation exists within the epithelial cells themselves. Regrettably, the other sensory modalities were not tested. This study suggests nerve-free *Hydr*a has a significantly impoverished sensory repertoire with much less access to the external world. It is no longer able to “see,” “taste,” or “smell,” and has a significantly reduced ability to “feel” mechanical stimulation.

### What kind of information integration/coordination?

After a *Hydra* body with sensory receptors is built, how is external information integrated with the animal’s own internal state and communicated organism-wide to create a unified whole? Is nerve-free *Hydra* less integrated than normal animals? While it is unknown how *Hydra*, which lacks a central nervous system, might integrate various external inputs with its own internal state, it has been hypothesized that a spontaneous low frequency neural network—rhythmic potential 1 (RP1)—might serve this function (Hanson 2021). Since the early work of Passano and McCullough it has been known that normal *Hydra* exhibits ongoing, rhythmic, spontaneous neural activity, even in the absence of behavior (Passano and McCullough 1962, 1963, 1965). What such “cryptic” neural activity is doing remains a mystery. In normal *Hydra*, RP1 is active even when the animal is at rest, and changes its firing frequency in accord with both the internal state of the animal and external input, suggesting it might serve as the ultimate integrator of electrical information in the system (see (Hanson 2021) for further review). Thus, in the single early study in which the electrophysiological properties of nerve-free *Hydra* were investigated, it was no surprise to find that normal *Hydra* “sporadically” produced spontaneous electrical potentials with a mean frequency of one per several minutes, while nerve-free animals failed to produce a single spontaneous electrical pulse in 10 h of recording from an extracellular electrode inserted into the body column of the animal (Campbell et al. 1976). While this is only one, relatively cursory, study, the result suggests the nervous system is required to generate spontaneous electrical activity in the animal. What such spontaneous electrical activity might be doing will be addressed further in the discussion section below.

In addition to the loss of spontaneous electrical activity in nerve-free animals, the speed of electrical communication in nerve-free *Hydra* was also significantly reduced (Campbell et al. 1976). When *Hydra* was placed in a chamber with recording electrodes in both its head and foot and a stimulating electrode at various points along its body column, the conduction velocity in normal tissue was 5.7 cm/s and in nerve-free tissue only 1.8 cm/s. Importantly, the conduction velocity was rescued when i-cells were transplanted back into nerve-free animals suggesting the slowed conduction was due to i-cell loss, not due to epithelial cell damage. This nearly five-fold decrease in electrical conduction in nerve-free *Hydra*, suggests information integration and propagation is significantly reduced in these animals. Electrical conduction in nerve-free tissue is not zero, however, indicating the epithelial tissue alone can still propagate electrical information body-wide, albeit at a significantly reduced rate. Electrical conduction is inhibited when nerve-free *Hydra* are treated with heptanol (a gap junction inhibitor), suggesting the electrical signal is mediated by gap junctions between epithelial cells (Takaku et al. 2014).

### What kind of behavior?

**Fig. 5:**
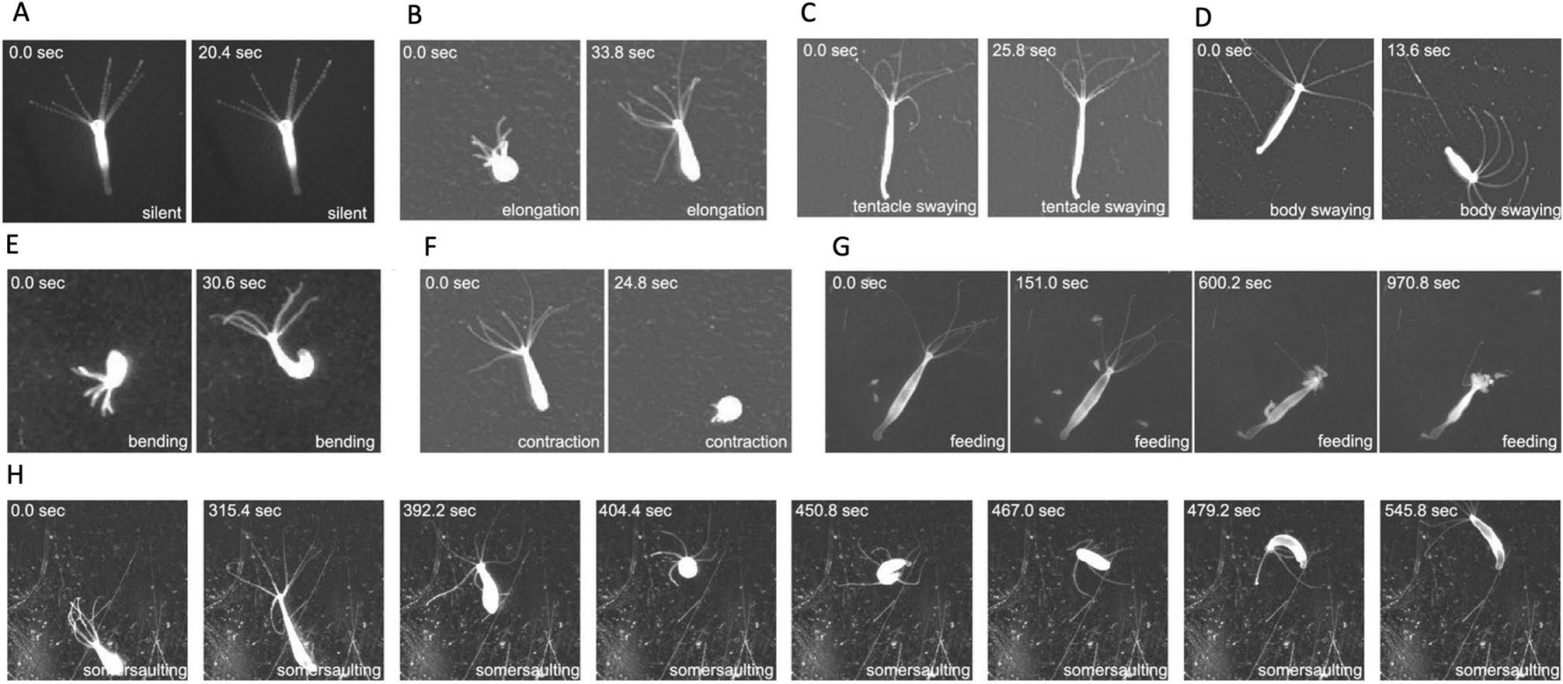
*Hydra* behavior. *Hydra* exhibits both simple and complex behaviors that have been quantified using machine learning (Han et al. 2018). *Hydra* also spends a significant amount of time “resting” or “silent,” not engaged in any behavior **A**. Simple *Hydra* behaviors include elongation **B**, tentacle swaying **C**, body swaying **D**, bending **E**, and full body contraction **F**. Complex *Hydra* behaviors include an elaborate feeding response **G** and somersaulting **H**. Figure adapted from Fig. 1E–L in (Han et al. 2018) (http://creativecommons.org/licenses/by/4.0/)

Once a *Hydra* body is built, sensors added, and external and internal information integrated, what kind of coordinated movement can be generated both with and without neurons? Despite possessing a simple nerve net, normal *Hydra* can perform some quite complex behaviors, including somersaulting end over end and moving like an inchworm (Leeuwenhoek 1703; Trembley et al. 1744; Passano and Mccullough 1963; Han et al. 2018) (Fig. 5). It also executes an elaborate feeding response in which it catches multiple prey (*Artemia* nauplii in the lab) with its tentacles, brings the captured prey towards its mouth (which opens with prey capture), and, finally, inserts the prey into its gastric cavity (Lenhoff 1961). In addition to these more complex movements, *Hydra* also performs several simple behaviors, including full body contraction, elongation, body swaying, bending, egestion, and tentacle swaying (Han et al. 2018). All these behaviors can occur spontaneously or in response to an external stimulus, except for the feeding response, which only occurs in response to food or a small-molecule stimulus (Loomis 1955; Lenhoff 1961).

When neurons are removed, most spontaneous behavior is lost, and the animal mostly lies motionless on its side as it can no longer attach its foot to the dish like normal *Hydra* (Campbell 1976; Campbell et al. 1976; Marcum and Campbell 1978a). In time-lapse movies, however, some spontaneous behavior was observed in nerve-free *Hydra*, including fluid expulsion from its mouth several times a day (Campbell et al. 1976; Marcum and Campbell 1978a). This causes collapse of the animal followed by re-extension, column pulsing, tentacle bending, and “annulations” (rings) along the body column. All these spontaneous movements were “more pronounced” after eating (Marcum and Campbell 1978a). Not only does nerve-free *Hydra* lack most spontaneous behavior, it also lacks most stimulus-evoked behavior, including the ability to detect, capture, and ingest prey (Campbell 1976; Campbell et al. 1976; Marcum and Campbell 1978a). Thus, nerve-free *Hydra* can only survive a few weeks in the lab unless it is manually force-fed shrimp, and subsequently “burped” by manually forcing the undigested particles out of its gastric cavity (Campbell 1976; Tran et al. 2017). Clearly, the nerve-free animals would not survive long in the wild, but with hand-feeding in the lab, they have been kept alive for over two years, and could likely be kept alive indefinitely if continually manually fed (Campbell 1976; Fradkin et al. 1978). While nerve-free *Hydra* no longer responds to food, light, or mild mechanical stimulation, it does respond to strong stimulation. Pinching nerve-free *Hydra* with forceps and direct electrical stimulation of the body column can both induce whole-body and tentacle contraction followed by re-extension of the animal (Campbell et al. 1976).

### What kind of learning and memory?

Having constructed a body that can sense, integrate, and move, what kinds of things can *Hydra* learn and remember both with and without neurons? Learning and memory are understudied in basal organisms, especially Cnidaria (see Cheng 2021 for a recent review). While very few studies have been published on this topic, normal *Hydra* have been shown to exhibit both habituation and sensitization. In 1905 Wagner showed normal *Hydra* stopped contracting after frequent repeated mechanical stimulation, but did not stop contracting when the interstimulus interval was increased (Wagner 1905). These results suggested *Hydra* habituated to the more frequent mechanical stimulus, but dishabituation was never formally tested so it was unclear if the animal simply became fatigued. These results were corroborated 50 years later by Rushforth and colleagues when they also found *Hydra* stopped contracting in response to repeated mechanical stimulation (Rushforth et al. 1963). In their study, however, dishabituation was demonstrated by showing the animal began contracting again when they switched to a light stimulus. These studies established that normal *Hydra* do exhibit habituation, the most rudimentary form of learning. In addition to habituation, Wagner showed normal *Hydra* are also capable of sensitization (Wagner 1905). After *Hydra* initially habituated to a repeated mechanical stimulus and stopped contracting, it subsequently responded to the same stimulus by exhibiting “escape” behavior in which it moved away from the site of stimulation. No studies on associative learning in normal *Hydra* have been published to date (Cheng 2021). At present, no studies on learning or memory have been published with nerve-free *Hydra.* Whether the ability of *Hydra* to both habituate and sensitize depends on neurons, or if epithelial animals might retain these capacities, remain interesting and open questions.

## Discussion: what nerve-free *hydra* suggest about neuronal function

**Fig. 6:**
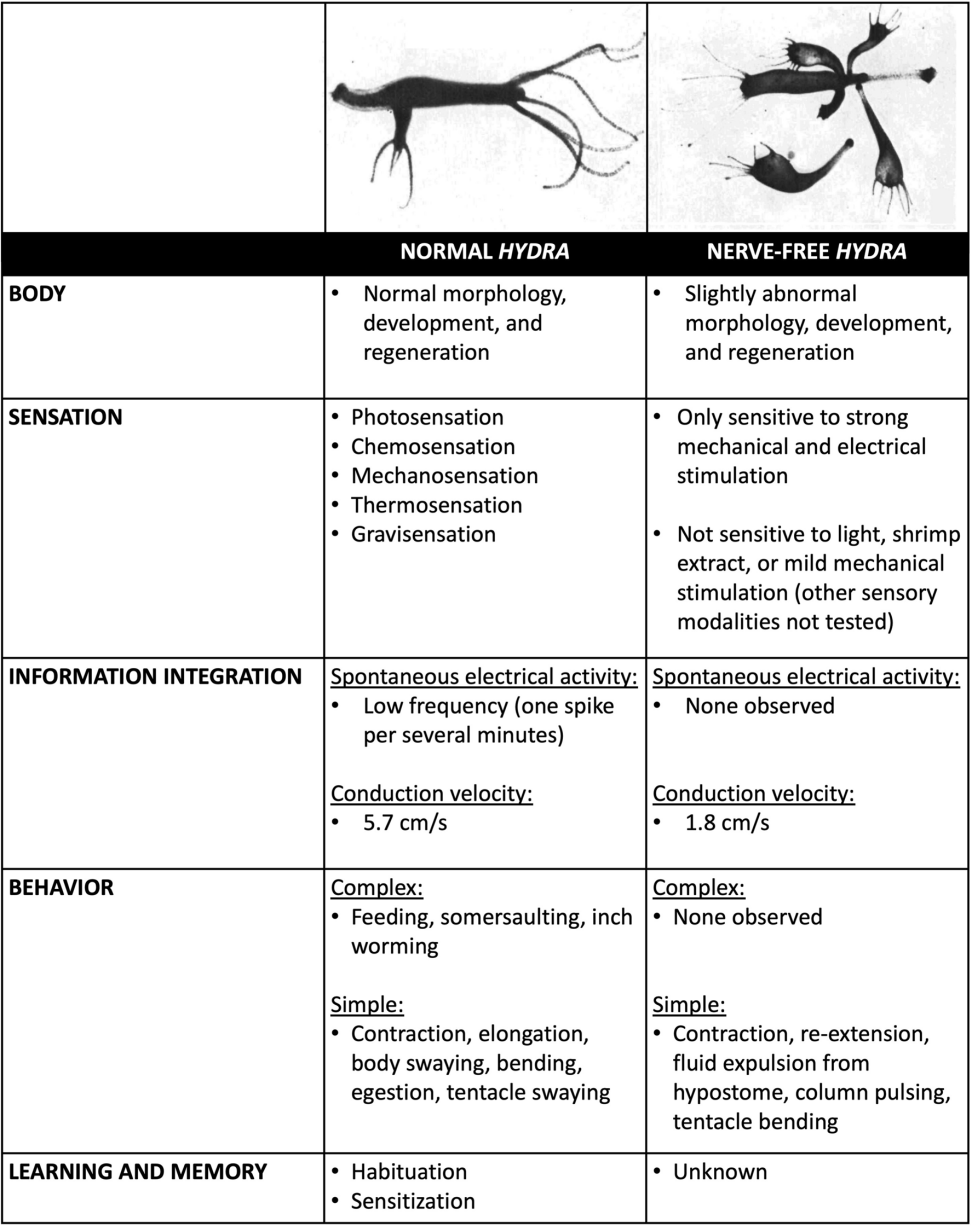
Summary of differences between *Hydra* with and without neurons. Top left figure shows the morphology of normal *Hydra* as compared to the morphology of nerve-free *Hydra* on the top right. Both figures adapted from Fig. 1A and 1G, respectively, with permission from (Campbell 1976)

Time to take stock. What do these findings in *Hydra* imply about the kind of animal that exists with and without neurons? Further, what do the discrepancies between the two kinds of animals (Fig. 6) tell us about the role of the nervous system in *Hydra*? Normal *Hydra* build reproducible tubular bodies with dome-shaped hypostomes and very tightly regulated numbers of tentacles and buds. These bodies have remarkable regenerative capacities that reliably recreate this structure efficiently and precisely. The construction of these bodies also involves the synthesis of sensors that allow the animal to see (Passano and McCullough 1962, 1965; Plachetzki et al. 2012), taste (Ewer and Fox 1947; Loomis 1955; Lenhoff 1961), touch (Mast 1903; Wagner 1905; Rushforth et al. 1963; Badhiwala et al. 2021), smell (Ewer and Fox 1947; Loomis 1955; Lenhoff 1961), detect temperature (Mast 1903; Schroeder and Callaghan 1981; Bosch et al. 1988; Tzouanas et al. 2021), and sense up from down (Mast 1903; Schroeder and Callaghan 1981; Bosch et al. 1988; Tzouanas et al. 2021), providing access to a rich external world. Internally, these animals have ongoing low-frequency spontaneous electrical activity, even while at rest, and conduct electrical information body-wide at a speed of 5.7 cm/s (Campbell et al. 1976). These low-frequency oscillations may serve both to integrate electrical information in the animal (Hanson 2021) and produce spontaneous and stimulus-evoked behaviors that allow the animal to capture prey, eat, locomote, contract, elongate, bend, and sway (Han et al. 2018). In addition to executing both simple and complex behaviors, these animals exhibit rudimentary learning in the form of habituating and sensitizing to noxious stimuli (at least) (Wagner 1905; Rushforth et al. 1963; Cheng 2021). Evidently, a *Hydra* with neurons is a rather sophisticated creature, capable of surviving indefinitely in favorable conditions.

When neurons are removed, however, a very different animal emerges (Fig. 6). This *Hydra’s* body is swollen, with a flat hypostome; short, irregularly spaced tentacles of variable number; a long skinny stalk; and foot no longer capable of adhering to its substrate. These bodies are awkward, variably shaped, with abnormal numbers of attached buds (Campbell 1976; Fradkin et al. 1978; Marcum and Campbell 1978a; Sugiyama and Fujisawa 1978a; Sacks and Davis 1979). While these bodies can regenerate, they do so more slowly and less precisely (Marcum and Campbell 1978a; Sacks and Davis 1979; Miljkovic-Licina et al. 2007). These animals also lack the sensory capacities of normal *Hydra* and can no longer see, taste, or feel light touch, but can sense strong mechanical and electrical stimulation (Campbell et al. 1976). Internally, they lack spontaneous electrical activity, but can conduct electrical information body-wide at 1.8 cm/s (Campbell et al. 1976). This lack of internal electrical activity correlates with a lack of most (but not all) spontaneous behavior (Campbell 1976; Campbell et al. 1976; Marcum and Campbell 1978a). These animals can still perform coordinated whole-body contractions when stimulated strongly but can no longer detect and capture prey and so cannot feed themselves (Campbell 1976; Campbell et al. 1976; Marcum and Campbell 1978a). It is unknown if these animals can learn or remember. Without being force-fed in the lab, nerve-free *Hydra* only survive a few weeks (Campbell 1976) and would certainly not last long in the wild. Clearly, these creatures are quite different than those that contain neural hardware.

### Building bodies

What do these findings tell us about the overall role of the nervous system in these animals? First, the picture is not entirely straightforward. These studies demonstrate the *Hydra* nervous system plays some role in the morphogenesis of the organism. While nerve-free *Hydra* can surprisingly build a near-normal body without neurons, they exhibit numerous morphological abnormalities, in addition to alterations in growth and budding rates (Marcum and Campbell 1978a; Sugiyama and Fujisawa 1978a; Sacks and Davis 1979), induction (Marcum and Campbell 1978a; Sugiyama and Fujisawa 1978a), regeneration (Marcum and Campbell 1978a; Sacks and Davis 1979; Miljkovic-Licina et al. 2007), and reversal of tissue polarity (Marcum et al. 1977). These aberrations are, however, mostly quantitative rather than qualitative. Thus, as was proposed in the 1970’s, it is likely the neurons are needed for “fine-tuning” or “patterning” the morphogenesis of the organism (Marcum and Campbell 1978a). Precisely how neurons might fine-tune the epithelial cells remains unknown. Two general possibilities exist: (i) by secreting molecules, and (ii) by influencing the non-neural bioelectric signaling in the epithelial cells that is known to be important for large-scale patterning of other species (Levin et al. 2017; Levin and Martyniuk 2018).

At the time nerve-free *Hydra* were first created, neurons were thought to produce a morphogenetic gradient along the body column to establish the apical and basal poles of the animal by secreting “morphogenetic substances” (Schaller et al. 1996). A model was proposed in which short-range molecules dubbed “head activator”/“foot activator” were thought to be inhibited by long-range molecules dubbed “head inhibitor”/“foot inhibitor” to establish the anterior–posterior axis. It was thought the activators were small neuropeptides and inhibitors were small molecules, all secreted by neurons. This model was never validated experimentally, so that over time, the role of neurons in secreting neuropeptides or small molecules to guide development was replaced by the discovery of the classical molecular morphogen gradients (e.g., Wnt, BMP (De Robertis 2008)) produced by epithelial cells (Hobmayer et al. 2000; Broun and Bode 2002; Reinhardt et al. 2004; Rentzsch et al. 2007; Lengfeld et al. 2009; Nakamura et al. 2011; Vogg et al. 2019). However, whether neurons play a role in secreting these molecules, or affecting their expression in epithelial cells, remains to be determined.

Interestingly, when neurons are removed, epithelial cells upregulate expression of neural-specific genes, including several “neurotransmission” genes, suggesting epithelial cells are highly plastic and can compensate for the loss of neurons in nerve-free *Hydra* (Wenger et al. 2016). These findings support the hypothesis that “proto-neural” epithelial cells may have operated as both mechanical effectors and pattern generators (Mackie 1970; Arendt 2008) and can regain this “multifunctional” state when neurons are removed. Whether this multifunctional state of epithelial cells in nerve-free *Hydra* resembles the function of proto-neural epithelial cells that existed over 500 million years ago, or is specific to this unique context, cannot be known. Nevertheless, it is an interesting model worth further investigation to determine what kinds of adaptations the nerve-free epithelia are capable of when neural hardware disappears.

In addition to potentially secreting morphogens to guide development, more recent work has demonstrated neurons also secrete anti-microbial peptides (AMPs), thereby regulating which microbes constitute the microflora (holobiont) of *Hydra* (Klimovich and Bosch 2018). While the history of developmental biology has largely focused solely on emergence of the host organism’s cells and tissues, it is becoming increasingly clear that most embryos consist of multiple species of microbes in addition to the host’s cells (Carrier and Bosch 2022). Thus, development entails the host organism’s cells in concert with its associated microbes, which often change throughout the course of morphogenesis. The microbiome of *Hydra* is quite dynamic during development, for example, consisting of a highly diverse community of microbes in newly hatched polyps (e.g., Bacteroidetes, β-proteobacteria, Actinobacteria), which becomes progressively less varied over time resulting in a stable configuration consisting mostly of *Curvibacter* in the adult animal (Franzenburg et al. 2013). When microbes are removed from *Hydra*, as in germ-free animals, significant developmental abnormalities are seen, such as reduced budding rate (Rahat and Dimentman 1982) and increased size (He and Bosch 2022), suggesting its symbionts are required for normal morphogenesis.

If neurons secrete AMPs, which shape *Hydra’s* microbial community, then the loss of neurons should alter the microbiome, and, consequently, the morphogenesis of the animal. Indeed, nerve-free *Hydra* exhibits a dramatic ten-fold change in the composition of its microbial community from β-proteobacteria to Bacteroidetes, which likely has important developmental consequences (Fraune et al. 2009). On top of secreting AMPs themselves, neurons may also affect the secretion of AMPs by epithelial cells as loss of neurons results in upregulation of AMPs within epithelial cells, suggesting neurons may normally inhibit epithelial cell AMP secretion (Wenger et al. 2016). It is worth noting that in all studies to date, nerve-free *Hydra* have been grown in *Hydra* media containing the antibiotic Rifampicin (Campbell 1976; Fradkin et al. 1978; Sugiyama and Fujisawa 1978a; Sacks and Davis 1979) because in the initial colchicine studies, nerve-free *Hydra* died after several weeks due to an apparent bacterial infection (Campbell 1976). Addition of Rifampicin to the *Hydra* media in these studies likely also altered the microbiome, which may have contributed to the altered morphology of the animals. Overall, then, the nervous system of *Hydra* appears to sculpt the composition of its resident microbes, which likely influences what kind of body ultimately gets built.

Along with secreting molecules to control developmental processes, neurons also likely contribute to the non-neural bioelectric signaling in the epithelial cells of the animal. Accumulating evidence supports an important role for non-neural bioelectric circuits in guiding morphogenesis (Levin et al. 2017; Levin and Martyniuk 2018). Nerve-free animals likely have significantly altered bioelectric signaling and may have to rely solely on slower conduction through gap junctions in cells comprising two-dimensional epithelial sheets (as will be further discussed in Sect. “Integration and coordination” below). It is possible the nervous system—which acts faster and more long-range via its neural processes—may guide and fine-tune the non-neural bioelectric signaling in the epithelial cells to help pattern the animal (Fields et al. 2020). How the nervous system impacts non-neural bioelectric signaling is virtually unknown, and is an area likely to yield important insights into how the nervous system might be guiding developmental processes “top-down” (Pezzulo and Levin 2016).

### Sensory repertoire

In addition to influencing morphogenesis, neurons are also clearly critical for functions traditionally associated with nervous systems: sensation, information integration, and behavioral coordination. *Hydra* with neurons have more access to their external world and can see (Passano and McCullough 1962, 1965; Plachetzki et al. 2012), taste (Ewer and Fox 1947; Loomis 1955; Lenhoff 1961), touch (Mast 1903; Wagner 1905; Rushforth et al. 1963; Badhiwala et al. 2021), smell (Ewer and Fox 1947; Loomis 1955; Lenhoff 1961), detect temperature (Mast 1903; Schroeder and Callaghan 1981; Bosch et al. 1988; Tzouanas et al. 2021), and sense up from down (Mast 1903; Schroeder and Callaghan 1981; Bosch et al. 1988; Tzouanas et al. 2021), whereas *Hydra* without neurons can no longer see, taste, or smell, and have a significantly reduced ability to detect mechanical stimulation (Campbell et al. 1976). It is important to note that nerve-free *Hydra* is not totally devoid of sensory input from the external world, however, as it does respond to strong mechanical stimulation. This finding implies epithelial cells possess mechanoreceptors themselves and can transduce a mechanical stimulus received in one part of the animal (e.g., the foot) into a body-wide electrical signal to generate a whole-body behavioral response (e.g., whole-body contraction). It remains unclear if the nerve-free epithelial tissue might also be sensitive to other external stimuli as only one study has been reported thus far in which no response to “bright light,” “mild mechanical stimulation,” or shrimp extract was detected, but the details of those methods are lacking so it is unclear how thoroughly those responses were tested (Campbell et al. 1976). In addition, no studies on sensitivity to temperature or gravity in nerve-free *Hydra* have been reported, so it remains unclear if epithelial tissue can detect either kind of external information.

Further delineating what types of sensory information nerve-free *Hydra* can detect will shed light not only on the kinds of external worlds pre-neural metazoans may have perceived, but also the non-neural mechanisms they may have employed. Sensory perception is ubiquitous in living systems, including non-neural organisms such as bacteria (Nara et al. 1991; Manson 1992; Grebe and Stock 1998; Lyon 2015; Persat et al. 2015), placozoa (Pearse 1989; Srivastava et al. 2008), sponges (Maldonado 2006; Leys et al. 2019), protists (Ginger et al. 2008), fungi (Corrochano et al. 2016; Aleklett and Boddy 2021), and plants (Chaiwanon et al. 2016), all of which sense various aspects of their external and internal environments. The non-neural mechanisms by which these organisms perceive their various stimuli are, for the most part, poorly understood. Nerve-free *Hydra* could contribute to this growing body of research in which the non-neural tissue could be probed to gain insight into both the kinds of stimuli to which it might be sensitive and the non-neural cellular and molecular mechanisms that might underly such sensitivities.

For now, the existing evidence indicates neurons provide *Hydra* with a more diverse set of sensory inputs that allows the animal to sense and respond to a much wider array of stimuli in its external environment. Whether each input is mediated by neurons or sensitive nematocytes remains an open question.

### Integration and coordination

It is unclear how external inputs are integrated with the internal state of the animal; however, spontaneous electrical low frequency oscillations (SELFOs) have been proposed as potential electrical information integrators in the system (see Hanson 2021 for further review). Such SELFOs might serve two main functions: (i) to integrate all the “bottom-up” electrical information in the system to generate a unified “self”/“world” model, and (ii) to coordinate the system “top-down” by serving as a “biological clock” that is sensitive to both internal and external stimuli. If this is the case, a *Hydra* with such a SELFO would thus possess an integrated self/world model and a biological clock that could keep all its otherwise autonomous parts (i.e., individual cells) coordinated in time to allow the organism to act as one, unified whole.

No such SELFO was found in the one electrophysiological study of nerve-free *Hydra* performed to date (Campbell et al. 1976), suggesting these animals may lack such an electrical information integrator and organism-wide coordinator. However, the epithelial cells of the animal may be able to compensate for the loss of neurons and generate spontaneous low frequency electrical activity, as is found in other non-neural species, like plants (Fromm and Lautner 2007; Masi et al. 2009; Baluška and Mancuso 2013; Canales et al. 2018) and fungi (Slayman et al. 1976; Olsson and Hansson 1995; Adamatzky 2018). The function of SELFOs in these species is also unknown, but a role in organism-wide integration and communication has been proposed (Baluška and Mancuso 2013; Canales et al. 2018; Adamatzky 2018). Further studies are needed to firmly establish whether nerve-free *Hydra* possesses any non-neural spontaneous low-frequency electrical activity as is observed in these other non-neural species. If no such spontaneous low frequency electrical activity is present in nerve-free *Hydra*, it may be these animals lack an ultimate electrical information integrator leading to a less coordinated animal, as will be discussed further below.

Along with generating spontaneous electrical activity, the nervous system provides much faster electrical conduction organism-wide, as evidenced by the 5.7 cm/s versus 1.8 cm/s conduction velocity in normal and nerve-free animals, respectively (Campbell et al. 1976). This likely allows much faster information propagation from sensors to the potential integrator (i.e., SELFO) and from the integrator (i.e., SELFO) to the effectors; in this case, epitheliomuscular cells. While animals without neurons can only conduct electrical information from neighbor-to-neighbor in two-dimensional epithelial sheets, likely through gap junctions (Lepault et al. 1980), animals with neurons can skip over many epithelial cells and transmit information more long-range via neural processes. In this way, animals with neurons can both receive and send electrical information from distal parts of the animal nearly five times faster than animals that rely solely on slow epithelial conduction. Thus, in addition to generating spontaneous electrical activity, which might serve as an organism-wide electrical information integrator and coordinator, neurons also seem to enhance speed of electrical information propagation throughout the animal.

These two features: spontaneous electrical activity, and increased speed of electrical conduction, likely enable much more efficient organism-wide coordination in *Hydra* with neurons. Nerve-free *Hydra* may still generate a SELFO, and still clearly conduct electrical information body-wide (Campbell et al. 1976), but the significantly reduced rate of information propagation likely limits the amount of whole animal coordination. This appears to be the case, as normal *Hydra* exhibit much more complex behavior than nerve-free animals. *Hydra* with neurons execute multiple complex movements, including inchworming, somersaulting, and an elaborate feeding response (Leeuwenhoek 1703; Trembley et al. 1744; Lenhoff 1961; Passano and Mccullough 1963; Han et al. 2018). Each of these behaviors requires the coordination of its various body parts in space over minutes-long timescales. In contrast, the most complex movement nerve-free *Hydra* displayed was a simple whole-body contraction in response to strong mechanical stimulation (Campbell et al. 1976). These findings indicate the slow epithelial electrical conduction velocity in nerve-free animals is sufficient to coordinate at least *some* simple whole-body behavior. To generate more complex whole-body movements in space over longer timescales, however, neurons appear to be required, at least in *Hydra*.

### Learning and memory

While very little work has been done on learning and memory in *Hydra*, the existing data suggest animals with neurons can habituate and sensitize to noxious stimuli (Wagner 1905; Rushforth et al. 1963; Cheng 2021) demonstrating these metazoans are capable of at least these elementary forms of learning. Whether normal *Hydra* is capable of more advanced forms of associative learning remains to be determined. To date, no studies on learning and memory in nerve-free *Hydra* have been published, so it is entirely unknown whether habituation and sensitization in these animals depends on neural hardware. Likewise, no studies on associative learning in nerve-free *Hydra* have been reported, so it remains to be seen whether epithelial tissue itself might be capable of this more advanced form of learning.

Despite the common conception that learning and memory depend on sophisticated nervous systems, there is ample evidence of both habituation and associative learning in numerous non-neural organisms, including bacteria, protists, fungi, and plants (Baluška and Levin 2016; van Duijn 2017). Thus, in addition to habituation and sensitization, it is likely that *Hydra* with neurons can perform associative learning, if appropriately tested. Moreover, it is likely that nerve-free *Hydra* can also exhibit some form of learning, such as habituation to strong mechanical stimulation, the one pronounced stimulus-evoked behavior reported in these animals (Campbell et al. 1976). Testing associative learning in nerve-free *Hydra* may be more challenging given their paucity of behavior, but with creative experimental setups these capacities can plausibly be assessed. Certainly, much more work needs to be done to determine what neurons may, or may not, add to the organism’s ability to learn and remember.

### Summary

Taken together, the work on nerve-free *Hydra* suggests the nervous system plays many roles in the animal, including contributing to the efficient and precise building of its body with its full complement of sensors; integrating its external inputs with its internal state; and generating and coordinating its movement through space and time. The role of the nervous system in learning and memory in these animals remains to be determined.

## Future directions

While the studies of nerve-free *Hydra* summarized here provide much insight into the potential role of the nervous system, relatively little work has been done in this area, the bulk of which was performed in the 1970’s with dated methods. This leaves many future experiments to be done using modern tools. One major advance would be the ability to generate nerve-free *Hydra* without eliminating i-cells, which produce nematocytes, gland cells, and germ cells in addition to neurons. To date, all methods used to produce nerve-free *Hydra* eliminate all four of these cell types (Bode et al. 1976; Campbell 1976; Fradkin et al. 1978; Sugiyama and Fujisawa 1978a; Sacks and Davis 1979), making it difficult to determine which cell types are contributing to which process. If a method could be developed to remove or replace *only* neurons in *Hydra*, the distinct role of the nervous system in these animals could be deciphered more clearly. Although the currently available techniques to eliminate neurons from *Hydra* are not specific to neurons, the ability to remove and replace the entire nervous system still provides a unique preparation worth further investigation with more advanced methods.

There are many new tools available today to study nerve-free *Hydra* in a more comprehensive manner than was possible in the 1970’s. In terms of the nervous system, there are now transgenic *Hydra* lines that allow visualization of both the structure (green fluorescent protein [GFP] expressed in all neurons (Siebert et al. 2019)) and function (fluorescent genetically encoded calcium indicator [GCaMP] expressed in all neurons (Dupre and Yuste 2017)) of the entire *Hydra* nervous system at single-cell resolution using light microscopy. Similarly, there are also transgenic *Hydra* lines that allow visualization of both the structure (GFP expressed in all ectodermal cells and red fluorescent protein [RFP] expressed in all endodermal cells (Glauber et al. 2013)) and function (GCaMP expressed in all ectodermal cells and RCaMP expressed in all endodermal cells (Szymanski and Yuste 2019)) of all epithelial cells of the animal at single-cell resolution with light microscopy. In addition to the structure and function of the epithelial cells, voltage dyes are now available that would allow visualization of the non-neural bioelectric signaling potentially occurring in the epithelial tissue of the animal (Levin et al. 2017).

Using these tools, numerous interesting questions can be asked in three main contexts: during nerve net removal, in nerve-free animals, and during nerve net reconstruction. For example, what happens to the structure and function of the nerve net while neurons are being removed from the animal over time and how does that correlate with changes in morphology, sensation, electrical activity, behavior, and learning at different time points when different numbers of neurons are present? Is there a critical number of neurons at which each aspect of the animal becomes abnormal or is there a more linear relationship between neural number and loss of the normal *Hydra* phenotype? What about when neurons are added back into the animal? Is there similarly a critical number of neurons required to rescue the abnormal epithelial phenotype in all aspects? Is the structure and function of the re-established nerve net the same as that found in the animal prior to removing its nervous system? These are just a few questions that can begin to be addressed.

The same kinds of questions can be asked about epithelial cells. Such as, what happens to the structure and function of epithelial cells as neurons are removed, while they are missing, and when they are replaced? Most intriguing is whether non-neural bioelectric signaling can be found in nerve-free *Hydra* using voltage dyes and how it might change in the presence and absence of neurons. These kinds of experiments could give important insights into how neurons might influence non-neural bioelectric signaling in *Hydra*. In addition to these studies, it is now possible to do whole animal single cell RNA sequencing (Siebert et al. 2019), opening the possibility of sequencing *Hydra* at different timepoints during neuron removal and replacement to determine how gene expression changes body-wide in epithelial tissue. This would yield important information about what kinds of molecular adaptations the epithelial tissue makes when neurons are removed and replaced and provide further insight into whether epithelial cells may return to a more multifunctional proto-neural state when neurons are missing (Mackie 1970; Arendt 2008).

In addition to studies of whole animals of a single strain, *Hydra* also allows two other kinds of unique experiments. First, *Hydra* permits the generation of chimeric animals via grafting (Fig. 2B). Thus, all the above experiments could be performed in both single strains and with chimeric *Hydra* composed of two different strains (Saffitz et al. 1972; Marcum and Campbell 1978b; Sugiyama and Fujisawa 1978b; Lee and Campbell 1979). At present, the number of strains is limited by the strains of currently available transgenic animals; however, transgenic animals of different strains could be created to allow multiple combinations. Even without transgenic animals, the ability to transplant a nervous system of one strain into epithelial tissue of another provides a unique opportunity to investigate how much the nervous system of one strain contributes to the overall phenotype of the animal in a more rigorous and quantitative way than has been done thus far.

Another remarkable feature of *Hydra* is its ability to rebuild itself after being dissociated into single cells (Gierer et al. 1972; Lovas and Yuste 2021). This unusual capacity allows the investigation of how a body gets built using epithelial cells only, epithelial cells in combination with neurons from the same strain, and with different combinations of epithelial cells and neurons from different strains. Reaggregation experiments could also be performed with all the transgenic animals listed above, allowing one to focus on different aspects of the animal from the structure and function of the nervous system to the structure and function of the epithelial cells. Additionally, the role of microbes (Klimovich and Bosch 2018) and non-neural bioelectric signaling (Levin et al. 2017; Levin and Martyniuk 2018) could also be probed in the context of building a *Hydra* from dissociated single cells both with and without neurons.

Clearly, there are many future directions for this work, many more than can be outlined here, that will provide exciting new insight into the myriad roles the nervous system might play in *Hydra*.

## Conclusion

What nervous systems add to animals remains unclear in large part because we don’t have access to the life forms that were present over 500 million years ago when neurons likely evolved. Fortunately, at least one extant organism, the small freshwater cnidarian *Hydra*, survives both the removal and replacement of its entire nervous system allowing direct observation of what kind of animal emerges both with and without neural hardware. To date, much has been learned from the early studies of nerve-free *Hydra*, mostly conducted in the 1970’s. The main results thus far indicate the nervous system plays many roles in the animal, including contributing to precisely building the *Hydra* body with its full complement of sensors, integrating external input with its internal state, and generating and coordinating movement. While this early work has been revealing, much future work remains to be done with modern methods to further determine the numerous roles of the nervous system in *Hydra*. Significant technological advancements have been made in the last half-century, and recently more particularly, including the ability to generate transgenic *Hydra* lines that have allowed whole-animal high-resolution imaging of its entire nerve net and epithelial tissue. These new tools, in combination with the ability to remove and replace neurons, provide a powerful platform to paint an even fuller picture of what neurons add to a living animal. Insights gained about the function of the primitive nerve net in *Hydra* are likely to illuminate the role of nervous systems in more complex organisms, including humans, as these fundamental processes are likely highly conserved across species.

## Data Availability

Not applicable.

## References

[CR1] Adamatzky A (2018). On spiking behaviour of oyster fungi Pleurotus djamor. Sci Rep.

[CR2] Aleklett K, Boddy L (2021). Fungal behaviour: a new frontier in behavioural ecology. Trends Ecol Evol.

[CR3] Anderson PAV, Bouchard C (2009). The regulation of cnidocyte discharge. Toxicon.

[CR4] Arendt D (2008). The evolution of cell types in animals: emerging principles from molecular studies. Nat Rev Genet.

[CR5] Badhiwala KN, Primack AS, Juliano CE, Robinson JT (2021). Multiple neuronal networks coordinate Hydra mechanosensory behavior. eLife.

[CR6] Baluška F, Levin M (2016). On having no head: cognition throughout biological systems. Front Psychol.

[CR7] Baluška F, Mancuso S (2013). Root apex transition zone as oscillatory zone. Front Plant Sci.

[CR8] Bode HR (1996). The interstitial cell lineage of hydra: a stem cell system that arose early in evolution. J Cell Sci.

[CR9] Bode HR, Flick KM, Smith GS (1976). Regulation of interstitial cell differentiation in Hydra attenuata. I. Homeostatic control of interstitial cell population size. J Cell Sci.

[CR10] Bosch TC, Krylow SM, Bode HR, Steele RE (1988). Thermotolerance and synthesis of heat shock proteins: these responses are present in Hydra attenuata but absent in Hydra oligactis. Proc Natl Acad Sci.

[CR11] Brien P, Reniers-Decoen M (1955). La signification des cellules interstitielles des hydres d’eau douce et le probleme de la reserve embryonnaire. Bull Biol Fr Belg.

[CR12] Broun M, Bode HR (2002). Characterization of the head organizer in hydra. Development.

[CR13] Browne EN (1909). The production of new hydranths in Hydra by the insertion of small grafts. J Exp Zool.

[CR14] Budd GE, Jensen S (2017). The origin of the animals and a ‘Savannah’ hypothesis for early bilaterian evolution. Biol Rev.

[CR15] Burkhardt P, Jékely G (2021). Evolution of synapses and neurotransmitter systems: the divide-and-conquer model for early neural cell-type evolution. Curr Opin Neurobiol.

[CR16] Burnett AL, Diehl NA (1964). The nervous system of hydra. I. types, distribution and origin of nerve elements. J Exp Zool.

[CR17] Buzgariu W, Al Haddad S, Tomczyk S (2015). Multi-functionality and plasticity characterize epithelial cells in Hydra. Tissue Barriers.

[CR18] Campbell RD (1967). Tissue dynamics of steady state growth in Hydra littoralis. II. Patterns of tissue movement. J Morphol.

[CR19] Campbell RD (1972). Statocyst lacking Cilia in the Coelenterate Corymorpha palma. Nature.

[CR20] Campbell RD (1976). Elimination by Hydra interstitial and nerve cells by means of colchicine. J Cell Sci.

[CR21] Campbell RD, Bode HR, Lenhoff HM (1983). Terminology for morphology and cell types. Hydra: research methods.

[CR22] Campbell RD, Josephson RK, Schwab WE, Rushforth NB (1976). Excitability of nerve-free Hydra. Nature.

[CR23] Canales J, Henriquez-Valencia C, Brauchi S (2018). The integration of electrical signals originating in the root of vascular plants. Front Plant Sci.

[CR24] Carrier TJ, Bosch TCG (2022). Symbiosis: the other cells in development. Development.

[CR25] Chaiwanon J, Wang W, Zhu J-Y (2016). Information integration and communication in plant growth regulation. Cell.

[CR26] Cheng K (2021). Learning in Cnidaria: a systematic review. Learn Behav.

[CR27] Clarkson SG, Wolpert L (1967). Bud morphogenesis in Hydra. Nature.

[CR28] Corrochano LM, Kuo A, Marcet-Houben M (2016). Expansion of signal transduction pathways in fungi by extensive genome duplication. Curr Biol.

[CR29] David CN, Campbell RD (1972). Cell cycle kinetics and development of Hydra attenuata: I. Epithelial cells. J Cell Sci.

[CR30] David CN, Gierer A (1974). Cell cycle kinetics and development of Hydra attenuata: III. Nerve and nematocyte differentiation. J Cell Sci.

[CR31] David CN, Murphy S (1977). Characterization of interstitial stem cells in hydra by cloning. Dev Biol.

[CR32] De Robertis EM (2008). Evo-devo: variations on ancestral themes. Cell.

[CR33] Dolph PJ, Ranganathan R, Colley NJ (1993). arrestin function in inactivation of g protein-coupled receptor rhodopsin in Vivo. Science.

[CR34] Dübel S, Hoffmeister SAH, Schaller HC (1987). Differentiation pathways of ectodermal epithelial cells in Hydra. Differentiation.

[CR35] Dupre C, Yuste R (2017). Non-overlapping neural Networks in Hydra vulgaris. Curr Biol.

[CR36] Ewer RF (1946). A response to gravity in young Hydra. Nature.

[CR37] Ewer RF, Fox HM (1947). On the functions and mode of action of the nematocysts of Hydra. Proc Zool Soc Lond.

[CR38] Fields C, Bischof J, Levin M (2020). Morphological coordination: a common ancestral function unifying neural and non-neural signaling. Physiology.

[CR39] Fradkin M, Kakis H, Campbell RD (1978). Effects of γ irradiation of Hydra: elimination of interstitial cells from viable Hydra. Radiat Res.

[CR40] Franzenburg S, Fraune S, Altrock PM (2013). Bacterial colonization of Hydra hatchlings follows a robust temporal pattern. ISME J.

[CR41] Fraune S, Abe Y, Bosch TCG (2009). Disturbing epithelial homeostasis in the metazoan Hydra leads to drastic changes in associated microbiota. Environ Microbiol.

[CR42] Fromm J, Lautner S (2007). Electrical signals and their physiological significance in plants. Plant Cell Environ.

[CR43] Gierer A, Berking S, Bode H (1972). Regeneration of Hydra from reaggregated cells. Nat New Biol.

[CR44] Ginger ML, Portman N, McKean PG (2008). Swimming with protists: perception, motility and flagellum assembly. Nat Rev Microbiol.

[CR45] Glauber KM, Dana CE, Steele RE (2010). Hydra. Curr Biol.

[CR46] Glauber KM, Dana CE, Park SS (2013). A small molecule screen identifies a novel compound that induces a homeotic transformation in Hydra. Development.

[CR47] Grebe TW, Stock J (1998). Bacterial chemotaxis: the five sensors of a bacterium. Curr Biol.

[CR48] Hadzi J (1909). Über das Nervensystem von Hydra. Arb Zool Inst Univ Wien.

[CR49] Han S, Taralova E, Dupre C, Yuste R (2018). Comprehensive machine learning analysis of Hydra behavior reveals a stable basal behavioral repertoire. Elife.

[CR50] Hanson A (2021). Spontaneous electrical low-frequency oscillations: a possible role in Hydra and all living systems. Philos Trans R Soc Lond B Biol Sci.

[CR51] He J, Bosch TCG (2022). Hydra’s lasting partnership with microbes: the key for escaping senescence?. Microorganisms.

[CR52] Hessinger DA, Lenhoff HM (1988). Biology of nematocysts.

[CR53] Hobmayer B, Rentzsch F, Kuhn K (2000). WNT signalling molecules act in axis formation in the diploblastic metazoan Hydra. Nature.

[CR54] Hufnagel LA, Kass-Simon G, Hessinger DA, Lenhoff HM (1988). 27-Functional anatomy of nematocyte innervation in battery cell complexes of the hydra tentacle. The biology of nematocysts.

[CR55] Hufnagel LA, Kass-Simon G, Lyon MK (1985). Functional organization of battery cell complexes in tentacles of Hydra attenuata. J Morphol.

[CR56] Jékely G, Keijzer F, Godfrey-Smith P (2015). An option space for early neural evolution. Phil Trans R Soc B.

[CR57] Kass-Simon G, Hessinger DA, Lenhoff HM (1988). 28-Towards a neuroethology of nematocyst discharge in the tentacles of Hydra. The Biology of nematocysts.

[CR58] Kass-Simon G, Scappaticci AA (2002). The behavioral and developmental physiology of nematocysts. Can J Zool.

[CR59] Keijzer F, van Duijn M, Lyon P (2013). What nervous systems do: early evolution, input–output, and the skin brain thesis. Adapt Behav.

[CR60] Klimovich AV, Bosch TCG (2018). Rethinking the role of the nervous system: lessons from the Hydra holobiont. BioEssays.

[CR61] Krupnick JG, Gurevich VV, Schepers T (1994). Arrestin-rhodopsin interaction. Multi-site binding delineated by peptide inhibition. J Biol Chem.

[CR62] Lee H-T, Campbell RD (1979). Development and behavior of an intergeneric chimera of Hydra (Pelmatohydra oligactis interstitial cells: Hydra attenuata epithelial cells). Biol Bull.

[CR63] Leeuwenhoek AV (1703) IV. Part of a letter from Mr Antony van Leeuwenhoek, F. R. S. concerning green weeds growing in water, and some animalcula found about them. Phil Trans R Soc 23:1304–1311. 10.1098/rstl.1702.0042

[CR64] Lengfeld T, Watanabe H, Simakov O (2009). Multiple Wnts are involved in Hydra organizer formation and regeneration. Dev Biol.

[CR65] Lenhoff HM (1961). Activation of the feeding reflex in Hydra littoralis. I. Role played by reduced glutathione and quantitative assay of the feeding reflex. J Gen Physiol.

[CR66] Lentz TL (1968). Primitive nervous systems.

[CR67] Lentz TL, Barrnett RJ (1965). Fine structure of the nervous system of Hydra. Am Zool.

[CR68] Lepault J, McDowall AW, Grimmelikhuijzen CJP (1980). Intercellular junctions in nerve-free Hydra. Cell Tissue Res.

[CR69] Levin M, Martyniuk CJ (2018). The bioelectric code: an ancient computational medium for dynamic control of growth and form. BioSystems.

[CR70] Levin M, Pezzulo G, Finkelstein JM (2017). Endogenous bioelectric signaling networks: exploiting voltage gradients for control of growth and form. Annu Rev Biomed Eng.

[CR71] Leys SP, Mah JL, McGill PR (2019). Sponge behavior and the chemical basis of responses: a post-genomic view. Integr Comp Biol.

[CR72] Loomis WF (1955). Glutathione control of the specific feeding reactions of Hydra. Ann NY Acad Sci.

[CR73] Lovas JR, Yuste R (2021). Ensemble synchronization in the reassembly of Hydra’s nervous system. Curr Biol.

[CR74] Lyon P (2015). The cognitive cell: bacterial behavior reconsidered. Front Microbiol.

[CR75] Mackie GO (1970). Neuroid conduction and the evolution of conducting tissues. Q Rev Biol.

[CR76] Mackie GO (1990). The elementary nervous system revisited1. Am Zool.

[CR77] Maldonado M (2006). The ecology of the sponge larva1. Can J Zool.

[CR78] Manson MD, Rose AH (1992). Bacterial motility and chemotaxis. Advances in microbial physiology.

[CR79] Marcum BA, Campbell RD (1978). Development of Hydra lacking nerve and interstitial cells. J Cell Sci.

[CR80] Marcum BA, Campbell RD (1978). Developmental roles of epithelial and interstitial cell lineages in hydra: analysis of chimeras. J Cell Sci.

[CR81] Marcum BA, Campbell RD, Romero J (1977). Polarity reversal in nerve-free hydra. Science.

[CR82] Masi E, Ciszak M, Stefano G (2009). Spatiotemporal dynamics of the electrical network activity in the root apex. Proc Natl Acad Sci USA.

[CR83] Mast SO (1903). Reactions to temperature changes in spirillum, hydra, and fresh-water planarians. Am J Physiol Legacy Content.

[CR84] Miljkovic-Licina M, Chera S, Ghila L, Galliot B (2007). Head regeneration in wild-type hydra requires de novo neurogenesis. Development.

[CR85] Nakamura Y, Tsiairis CD, Özbek S, Holstein TW (2011). Autoregulatory and repressive inputs localize Hydra Wnt3 to the head organizer. Proc Natl Acad Sci.

[CR86] Nara T, Lee L, Imae Y (1991). Thermosensing ability of Trg and Tap chemoreceptors in Escherichia coli. J Bacteriol.

[CR87] Olsson S, Hansson BS (1995). Action potential-like activity found in fungal mycelia is sensitive to stimulation. Naturwissenschaften.

[CR88] Parker GH (1919). The Elementary nervous system.

[CR89] Passano LM, McCullough CB (1962). The light response and the rhythmic potentials of hydra. Proc Natl Acad Sci USA.

[CR90] Passano LM, Mccullough CB (1963). Pacemaker hierarchies controlling the behaviour of hydras. Nature.

[CR91] Passano LM, Mccullough CB (1965). Co-ordinating systems and behaviour in hydra. II. The rhythmic potential system. J Exp Biol.

[CR92] Pearse VB (1989). Growth and behavior of trichoplax adhaerens: first record of the phylum placozoa in Hawaii. Pac Sci.

[CR93] Persat A, Nadell CD, Kim MK (2015). The mechanical world of bacteria. Cell.

[CR94] Pezzulo G, Levin M (2016). Top-down models in biology: explanation and control of complex living systems above the molecular level. J R Soc Interface.

[CR95] Plachetzki DC, Fong CR, Oakley TH (2010). The evolution of phototransduction from an ancestral cyclic nucleotide gated pathway. Proc Biol Sci.

[CR96] Plachetzki DC, Fong CR, Oakley TH (2012). Cnidocyte discharge is regulated by light and opsin-mediated phototransduction. BMC Biol.

[CR97] Rahat M, Dimentman C (1982). Cultivation of bacteria-free Hydra viridis: missing budding factor in nonsymbiotic hydra. Science.

[CR98] Reinhardt B, Broun M, Blitz IL, Bode HR (2004). HyBMP5-8b, a BMP5-8 orthologue, acts during axial patterning and tentacle formation in hydra. Dev Biol.

[CR99] Rentzsch F, Guder C, Vocke D (2007). An ancient chordin-like gene in organizer formation of Hydra. Proc Natl Acad Sci.

[CR100] Rushforth NB, Burnett AL, Maynard R (1963). Behavior in Hydra: contraction responses of hydra pirardi to mechanical and light stimuli. Science.

[CR101] Sacks PG, Davis LE (1979). Production of nerveless Hydra attenuata by hydroxyurea treatments. J Cell Sci.

[CR102] Saffitz JE, Burnett AL, Lesh GE (1972). Nervous system transplantation in hydra. J Exp Zool.

[CR103] Scappaticci AA, Kahn F, Kass-Simon G (2010). Nematocyst discharge in Hydra vulgaris: differential responses of desmonemes and stenoteles to mechanical and chemical stimulation. Comp Biochem Physiol A Mol Integr Physiol.

[CR104] Schaller HC, Hermans-Borgmeyer I, Hoffmeister SA (1996). Neuronal control of development in hydra. Int J Dev Biol.

[CR105] Schroeder LA, Callaghan WM (1981). Thermal tolerance and acclimation of two species of Hydra1. Limnol Oceanogr.

[CR106] Siebert S, Farrell JA, Cazet JF (2019). Stem cell differentiation trajectories in Hydra resolved at single-cell resolution. Science.

[CR107] Slayman CL, Scott Long W, Gradmann D (1976). “Action potentials” in Neurospora crassa, a mycelial fungus. Biochimica Biophysica Acta.

[CR108] Srivastava M, Begovic E, Chapman J (2008). The Trichoplax genome and the nature of placozoans. Nature.

[CR109] Sugiyama T, Fujisawa T (1978). Genetic analysis of developmental mechanisms in hydra. II. Isolation and characterization of an interstitial cell-deficient strain. J Cell Sci.

[CR110] Sugiyama T, Fujisawa T (1978). Genetic analysis of developmental mechanisms in hydra. V. Cell lineage and development of chimera Hydra. J Cell Sci.

[CR111] Szczepanek S, Cikala M, David CN (2002). Poly-γ-glutamate synthesis during formation of nematocyst capsules in Hydra. J Cell Sci.

[CR112] Szymanski JR, Yuste R (2019). Mapping the whole-body muscle activity of Hydra vulgaris. Curr Biol.

[CR113] Takaku Y, Hwang JS, Wolf A (2014). Innexin gap junctions in nerve cells coordinate spontaneous contractile behavior in Hydra polyps. Sci Rep.

[CR114] Terada H, Sugiyama T, Shigenaka Y (1988). Genetic analysis of developmental mechanisms in hydra. XVIII. Mechanism for elimination of the interstitial cell lineage in the mutant strain Sf-1. Dev Biol.

[CR115] Tran CM, Fu S, Rowe T, Collins E-MS (2017). Generation and Long-term Maintenance of Nerve-free Hydra. J Vis Exp.

[CR116] Trembley A, Pronk C, Schley J van der, Lyonet P (1744) Mémoires pour servir à l’histoire d’un genre de polypes d’eau douce, à bras en forme de cornes. Chez Jean & Herman Verbeek, A Leide

[CR117] Tzouanas CN, Kim S, Badhiwala KN (2021). Hydra vulgaris shows stable responses to thermal stimulation despite large changes in the number of neurons. iScience.

[CR118] van Duijn M (2017). Phylogenetic origins of biological cognition: convergent patterns in the early evolution of learning. Interface Focus.

[CR119] Vogg MC, Beccari L, Iglesias Ollé L (2019). An evolutionarily-conserved Wnt3/β-catenin/Sp5 feedback loop restricts head organizer activity in Hydra. Nat Commun.

[CR120] Wagner G (1905). Memoirs: on some movements and reactions of Hydra. J Cell Sci.

[CR121] Watson GM, Hessinger DA (1989). Cnidocyte mechanoreceptors are tuned to the movements of swimming prey by chemoreceptors. Science.

[CR122] Webster G (1971). Morphogenesis and pattern formation in hydroids. Biol Rev.

[CR123] Wenger Y, Buzgariu W, Galliot B (2016). Loss of neurogenesis in Hydra leads to compensatory regulation of neurogenic and neurotransmission genes in epithelial cells. Phil Trans R Soc B.

[CR124] Wilson EB (1891). The Heliotropism of Hydra. Am Nat.

[CR125] Wood RL (1979). The fine structure of the hypostome and mouth of hydra. II. Transmission electron microscopy. Cell Tissue Res.

